# Genetic landscape of Chinese colorectal cancer: insights into germline and somatic mutations

**DOI:** 10.1186/s12885-026-15626-x

**Published:** 2026-03-10

**Authors:** Fazhi Zhao, Hexin Lin, Rui Han, Meng Wang, Lin Yang, Dianfeng Tian, Liangqi Zhong, Pengxin Zhang, Queling Liu

**Affiliations:** 1 Department of Gastric Surgery, Sichuan Clinical Research Center for Cancer, Sichuan Cancer Hospital and Institute, Sichuan Cancer Center, Affiliated Cancer Hospital of University of Electronic Science and Technology of China, Chengdu, Sichuan Province 610041 China; 2 Department of Gastrointestinal Oncology Surgery, School of Medicine, The First Affiliated Hospital of Xiamen University, Xiamen University, Xiamen, Fujian 361001 China; 3 Department of Pathology, Peking University First Hospital, Beijing, 100034 China; 4 Center of Gastrointestinal and Minimally Invasive Surgery, Department of General Surgery, The Third People’s Hospital of Chengdu, Chengdu, 610031 China; 5 Department of urology, Aerospace center hospital, Beijing, 100049 China; 6 Department of Colorectal Surgery, First Hospital of Shanxi Medical University, Taiyuan, Shanxi 030001 China; 7 Shanghai Tongshu Biotech Co Ltd, Shanghai, 201900 China; 8 Department of Pathology, The First Affiliated Hospital of Dalian Medical University, Dalian, Liaoning Province 116011 China; 9 Department of Oncology, The Second Affiliated Hospital of Nanchang University, Nanchang, Jiangxi 330000 China; 10 Department of Oncology, Jiangxi Provincial People’s Hospital, The First Affiliated Hospital of Nanchang Medical College, Nanchang, Jiangxi 330000 China

**Keywords:** Colorectal cancer, Germline mutations, Hereditary cancer, MMR, Next-generation sequencing

## Abstract

**Background:**

Germline pathogenic alleles predispose carriers to malignancies; failure to recognize the underlying cancer susceptibility and subsequent delayed diagnosis may lead to severe health impairment. It remains largely undetermined what the somatic mutation characteristics are in colorectal cancer (CRC) patients with pathogenic/likely pathogenic (P/LP) germline mutations and whether, and how, these germline mutations typically located in non-mismatch repair genes, are associated with CRC tumorigenesis.

**Methods:**

From 8,676 Chinese patients with CRC, we initially screened those who had undergone both germline and somatic mutation testing. We analyzed the relationship between germline mutations and the somatic mutational landscape in this cohort. Germline alterations were examined with a 556- or 105- gene next-generation sequencing panel, and somatic alterations were examined with a 556-gene panel.

**Results:**

Overall, 80 CRC patients were identified as carriers of P/LP germline mutations. After excluding 6 patients lacking somatic mutation testing data, 74 patients with P/LP germline mutations and 100 patients without germline mutations were finally enrolled in this study. Twenty-one germline genes were detected in the 74 patients. Compared with patients without P/LP mutations (non-P group), those in the P/LP mutations (P group) had significantly lower age and a higher ratio of microsatellite instability. Patients harboring P/LP germline mutations in MMR genes (P-MMR) were significantly younger and more frequently exhibited high tumor mutational burden. The most frequent somatic variations were *KRAS* and *TP53*. The somatic mutational landscape revealed a significantly higher mutational frequency in the P group than in the non-P group, and in the P-MMR group than in the P-non-MMR group. The MAPK pathway was significantly enriched in the P and P-MMR groups. Our data showed lower mutation rate of *MSH2* among all MMR genes than in the Western population. Population-based risk analysis indicated that the odds ratio of *BRCA2* and *ATM* mutations exceeded 10.

**Conclusions:**

Our findings offer a comprehensive overview of genetic susceptibility in Chinese CRC, which may help to shape a more comprehensive understanding of genetic structure of CRC and generate accurate individualized risk management strategies for mutation carriers.

**Supplementary Information:**

The online version contains supplementary material available at 10.1186/s12885-026-15626-x.

## Introduction

Colorectal cancer (CRC) is one of the most common neoplastic diseases worldwide, ranks third in cancer incidence and second concerning cancer-related mortality [1]. Inherited predisposition accounts for approximately one-third of CRC susceptibility [2, 3] and carriers of relevant mutations exhibit an increased risk of CRC [4–6]. Approximately 5–10% of CRCs are associated with high-risk mutations in known CRC susceptibility genes, predominantly the mismatch repair (MMR) genes (Lynch Syndrome, LS) [7], APC gene (Familial Adenomatous Polyposis, FAP) [8], MUTYH gene (MUTYH-associated polyposis, MAP) [9], and other syndromes. Except these hotspot mutations found in inherited CRC syndromes, new suspected inherited germline mutations have also been reported in recent years [7, 10, 11]. From 2003 to 2023, previous studies have primarily assessed the prevalence of hereditary CRC syndromes in Western countries, focusing on specific populations with certain syndromes, and sequencing limited gene panels associated with hereditary CRC [12–15]. Although analyses have been conducted recently on Chinese populations, the relevant research is still limited.

Precision medicine and tumor genomic profiling are being increasingly performed for cancer patients. These efforts have the potential to direct therapy, predict response, and define prognosis. With the advent of next-generation sequencing (NGS) technology, multigene panel testing is now widely used tool for screening hereditary CRC. Identifying people at high risk for CRC, especially those carrying CRC susceptibility gene mutations, is important to provide a variety of risk management options and targeted screening for cancer prevention [16]. Nonetheless, the precise magnitude of this risk in Chinese CRC patients has yet to be definitively characterized. CRCs with distinct germline mutation statuses may originate from divergent tumorigenic pathways and display heterogeneous somatic mutational profiles. Understanding the connections of germline and somatic mutations in CRC patients is critical to elucidating the genetic pattern, helping confirm germline tumorigenic effects and identifying potential therapeutic targets.

In this study, we identified CRC-predisposing germline mutations and performed extensive comparisons between patients with and without P/LP germline mutations among 174 Chinese CRC patients. We aimed to investigate their potential influences on CRC somatic mutational features and recognized distinct germline tumorigenic patterns. This may help to shape a more comprehensive understanding of genetic structure of CRC and generate accurate individualized risk management strategies for mutation carriers.

## Materials and methods

### Study samples

We retrospectively enrolled 8,676 unselected CRC patients who had undergone genetic testing. All enrolled patients provided matched white blood cell samples, which were used for germline mutation calling, including the identification of pathogenic/likely pathogenic (P/LP) germline variants. Patients with pathogenic/likely pathogenic (P/LP) germline mutations were informed of the test results. Among the enrolled patients who had undergone both somatic and germline mutation testing, 80 patients were identified as carrying P/LP germline variants. Six of these 80 patients were excluded due to the absence of somatic mutation data. To enable balanced comparison between patients with and without P/LP germline mutations, 100 patients who do not carry P/LP germline mutations were randomly selected. Patients were enrolled in accordance with the following inclusion criteria: (i) Histologically confirmed CRC by two independent pathologists via hematoxylin and eosin (H&E) staining; (ii) Availability of next-generation sequencing (NGS) data from formalin-fixed paraffin embedded (FFPE) tissues and paired white blood cell samples obtained at the time of CRC diagnosis. A total of 174 CRC patients were ultimately selected from participating hospitals, and the selection was based on the availability of both somatic mutation data and germline mutation data from these patients. Those who met the above criteria were included in this study, with no further selection criteria applied. This study was conducted according to the guidelines of the Declaration of Helsinki. It was based on retrospective, de-identified clinical data and received a waiver of patient consent from the Institutional Review Board.

### DNA extraction and sequencing

Tissue samples were used for tumor DNA detection and leucocyte samples for germline DNA detection. The QIAamp DNA FFPE tissue kit (Qiagen, No. 56404, Valencia, CA, USA) was employed to extract genomic DNA from FFPE tissues. Additionally, matched white blood cell DNA for germline testing were extracted using the DNeasy Blood & Tissue kit (Qiagen, No. 69504, Valencia, CA). Both cancer tissue and white blood cell genomic DNA were quantified with a Qubit 2.0 fluorometer and Qubit dsDNA HS assay kit (Thermo Fisher Scientific, Inc., Waltham, MA, USA) in accordance with the manufacturer’s instructions. Libraries were prepared from extracted DNA (≥ 100ng DNA from tissues; 20ng DNA from blood) and sequenced on Illumina NovaSeq 6000 platform (Illumina Corporation, Illumina, San Diego, CA, USA) in 100 bp paired-end mode with a minimum coverage of 1000 X. The libraries were subjected to enrichment using multi-gene panels (556- or 105- cancer related gene panel) developed by Shanghai Tongshu Biotechnology Co., Ltd. The Burrows-Wheeler Aligner (BWA, http://bio-bwa.sourceforge.net/) software was utilized to align the clean paired-end reads to the human genome build the GRCh37 / hg19. The median effective sequencing depth of germline DNA was 3422× (range, 297–16180×). The median effective sequencing depths of tumor DNA were 2473× (range, 827–5876×) in the tumor tissue cohort. For alignment optimization, variant calling, and annotation, we utilized GATK [17], MuTect [17], and VarScan [18], respectively. GATK (version 3.6; haplotype caller in single-sample mode with duplicate and unmapped reads removed using default parameters) was used to detect single-nucleotide variants (SNVs) and small insertions and deletions (indels) from germline DNA samples. Somatic mutations and indels were called through MuTect (version 1.1.7; http://www.broadinstitute.org/cancer/cga/mutect) and VarScan 2 (v2.4.3; http://varscan.sourceforge.net/) and annotated using ANNOVAR (version 20191024; http://www.openbioinformatics.org/annovar/). The copy number variation (CNV) was detected with CNVkit version 0.9.3 (https://github.com/etal/cnvkit). Germline variants were classified per the Ambry five-tier variant classification protocol, including pathogenic mutation; variant, likely pathogenic; variant of unknown significance; variant, likely benign; and benign, which is based on guidelines published by the American College of Medical Genetics and Genomics (ACMG), the Association for Molecular Pathology, and the International Agency for Research on Cancer [19, 20]. As a result, all germline mutations were categorized as pathogenic/likely pathogenic (defined P group) or non-pathogenic/likely pathogenic (defined non-P group). MutationMapper (https://www.cbioportal.org/mutation_mapper) was used and modified to simultaneously visualize genetic lesions [21].

### Tumor mutational burden (TMB) and microsatellite instability (MSI) analysis

TMB was calculated by using an algorithm as somatic mutation of genomic detection, including coding base substitutions and indel mutation per megabase (muts/Mb) as previously reported [22]. TMB-high (TMB-H) was defined as TMB ≥ 6.6 muts/Mb, and whereas a TMB < 6.6 muts/Mb was considered low (TMB-L). MSI status determination involved scanning the MSI loci within the reference genome, and read counts aligned to loci with different repeat lengths were quantified using a training set of samples with known MSI status. Read counts at each repeat length were calculated using a strategy similar to that proposed by MSIsensor. MSIsensor was employed to classify CRCs into MSI-H (microsatellite instability high; MSIsensor score ≥ 10) and MSI-L/MSS (microsatellite instability low or microsatellite stable; MSIsensor score < 10) cases [23–26].

### Kyoto Encyclopedia of Genes and Genomes (KEGG) and gene ontology (GO) analysis

The GO and KEGG functional enrichment analysis of mutated genes was conducted utilizing the ClusterProfiler R package. Adjusted p-values were calculated using the Benjamini–Hochberg method, with statistical significance established at an adjusted p-value threshold of < 0.05. The results of the enrichment analyses were visualized using the ggplot2 R package.

### Mutational signatures

We utilized non-negative matrix factorization from the R package NMF to analyze the mutational signatures. The base substitutions were categorized into 6 directions (C > T, C > A, C > G, T > C, T > G, and T > A). By applying the NMF algorithm, we deconstructed the mutational signatures. Cosine similarity was employed as a metric to assess the resemblance between our signatures and those in COSMIC.

### Statistical analysis

Statistical analyses were carried out using the R version 3.4.4 software (The R Foundation of Statistical Computating). The Chi-square test and Fisher’s exact test were used to compare differences in categorical parameters between different groups. The Mann-Whitney U test was used to analyze variations in continuous variables among the groups. All variables included age, sex, germline mutation status were included in the univariable and multivariable logistic regression analyses. The odds ratio (OR) was determined by comparing the frequency of a specific germline mutation in the general population sourced from the Genome Aggregation Database (gnomAD) with the mutation frequency identified in this study. False discovery rate (FDR) correction was done by the Benjamini-Hochberg method. All *P* values were two-sided, and *P* values of < 0.05 were considered statistically significant.

## Results

### Patient characteristics

Among patients with pathologically confirmed CRC who underwent genetic testing, a total of 174 patients (74 P and 100 non-P) with NGS data and relatively complete clinical information were included (Table 1). Of these patients, approximately 67.82% (118/174) were at least 50 years old, and 59.77% (104/174) cases were male. Additionally, 38.51% (67/174) patients had TMB-H and 18.39% (32/174) patients exhibited MSI-H.

**Table 1:** Clinical characteristics of the 174 Chinese CRC patients who underwent hereditary genetic testing

Characteristic	Group *P* (%)	Group Non-*P* (%)	*P* value
Total	74 (42.53%)	100 (57.47%)	
Age			**0.0352**
< 50 years	27 (36.49%)	24 (24.00%)	
≥ 50 years	42 (56.76%)	76 (76.00%)	
Unknown	5 (6.76%)	0 (0.00%)	
Gender			0.0514
Female	36 (48.65%)	34 (34.00%)	
Male	38 (51.35%)	66 (66.00%)	
TMB level			0.8762
TMB-high	28 (37.84%)	39 (39.00%)	
TMB-low	46 (62.16%)	61 (61.00%)	
MSI status			**0.0034**
MSI-H	21 (28.38%)	11 (11.00%)	
MSS	53 (71.62%)	89 (89.00%)	

Note: Boldface indicate statistical significance

*P* pathogenic, *non-P* non-pathogenic, *MSI* microsatellite instability, *MSI-H* microsatellite instability-high, *MSS* microsatellite stable, *TMB* tumor mutation burden

### Molecular and clinical characteristics of patients with P/LP germline variants

**Fig. 1:**
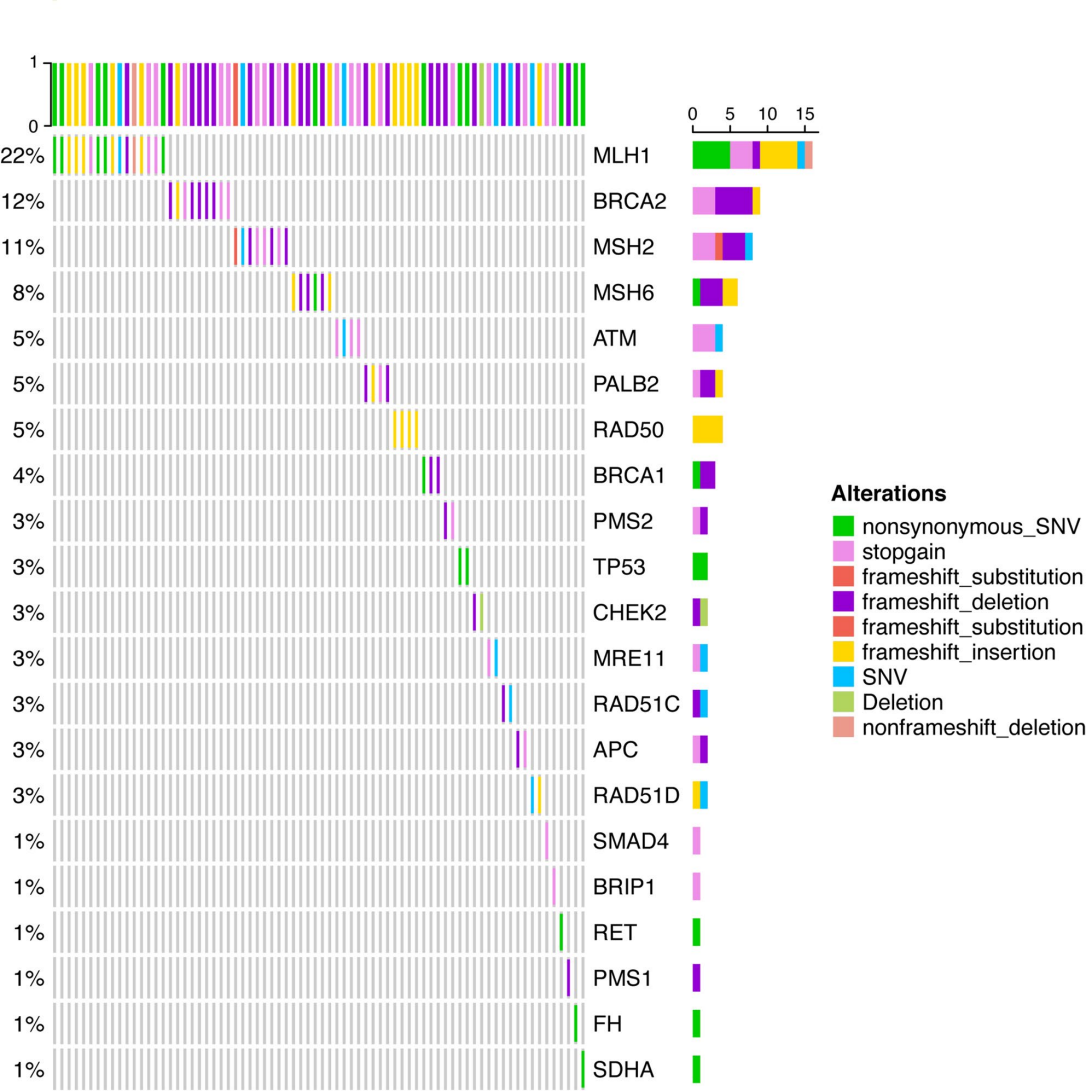
Repertoire of germline genetic alterations of colorectal cancer in the present cohort

A total of 74 distinct P/LP germline variants were identified in 74 CRC patients with P/LP germline mutations, and each of these patients carried exactly one germline variant (Fig. 1). Germline mutations were identified in 21 genes, with the top five most frequently genes being *MLH1* (21.6%), *BRCA* (12.2%), *MSH2* (10.8%), *MSH6* (8.1%), and *ATM* (5.4%) (Fig. 1, Figure S1A). MutationMapper analysis revealed that in addition to *BRCA2*, the mutation sites of the other three high-frequency mutation genes were *MLH1*, *MSH2*, and *MSH6* (Figure S1B-E). We further analyzed the age distribution at diagnosis of patients carrying different germline mutations. The results showed that the age at diagnosis in patients with *MLH1* germline mutation was significantly lower than in patients with *BRCA2*, *MSH2*, and *MSH6* germline mutation (*P* = 0.025, 0.026, 0.006, respectively, Figure S2). As for copy number variation (CNV) and MSI status, we analyzed them in patients harboring different germline gene mutations. Our results demonstrated that patients with *BRCA1* germline mutations had a higher proportion of detectable CNVs (*P* = 0.046, Figure S3), and those with *MLH1* germline mutations exhibited a significantly higher proportion of MSI-H (*P* = 0.010, Figure S3).

Patients were stratified into two groups based on the presence or absence of P/LP germline mutations: the P group (patients harboring P/LP germline variants) and the non-P group (patients without P/LP germline mutations). Compared with the non-P group, the P group had a significantly higher ratio of patients aged < 50 years (36.49% vs. 24.00%, *P* = 0.0352%, Table 1**)** and a lower median age (53.5 vs. 59, *P* = 0.0094, Figure S4A). The proportion of female patients was higher in the P group than that in the non-P group, though this difference did not reach statistical significance (48.65% vs. 34.00%, *P* = 0.0514, Table 1, Figure S4B). No significant difference was observed in the proportion of patients with TMB-H between the P group and the non-P group (37.84% vs. 39.00%, *P* = 0.8762, Table 1, Fig. S4C). However, the proportion of MSI-H patients was significantly higher in the P group than that in the non-P group (28.38% vs. 11.00%, *P* = 0.0034, Table 1, Fig. S4D).

Furthermore, we observed an association between common germline mutations and MMR. With the mutation statuses of four genes (*MLH1*, *MSH2*, *MSH6*, and *PMS2*) available in our cohort, we further investigated the clinical characteristics of patients with germline MMR mutations (P-MMR group) and patients with non-MMR germline mutations (P-non-MMR group). Patients with germline MMR gene mutations tended to have a higher ratio of patients aged < 50 years (45.45% vs. 29.27%, *P* = 0.1047, Table 2) and a lower median age (48 vs. 58, *P* = 0.062, Fig. S5A) than those with germline non-MMR gene mutations, without finding statistical differences. The proportion of female patients was higher in the P-MMR group than that in the P-non-MMR group, though this difference did not reach statistical significance (60.61% vs. 39.02%, *P* = 0.0648, Table 2, Fig. S5B). Interestingly, the ratio of TMB-H (60.61% vs. 19.51%, *P* = 0.0003, Table 2, Fig. S5C) and MSI-H (54.55% vs. 7.32%, *P* < 0.0001, Table 2, Fig. S5D) patients was significantly higher in the P-MMR group than those in the P-non-MMR group. Additionally, the P-MMR group had a significantly lower proportion of CNV detectable (*P* = 0.002, Fig. S3C), whereas no significant difference was observed between two groups (*P* = 0.673, Fig. S3D).

**Table 2:** Clinical characteristics of the 74 Chinese CRC patients with pathogenic germline mutations

Characteristic	Group *P*-MMR	Group *P*-Non-MMR	*P* value
Total	33 (44.59%)	41 (55.41%)	
Age			0.1047
< 50 years	15 (45.45%)	12 (29.27%)	
≥ 50 years	15 (45.45%)	27 (65.85%)	
Unknown	3 (9.09%)	2 (4.88%)	
Gender			0.0648
Female	20 (60.61%)	16 (39.02%)	
Male	13 (39.39%)	25 (60.98%)	
TMB level			**0.0003**
TMB-high	20 (60.61%)	8 (19.51%)	
TMB-low	13 (39.39%)	33 (80.49%)	
MSI status			**< 0.0001**
MSI-H	18 (54.55%)	3 (7.32%)	
MSS	15 (45.45%)	38 (92.68%)	

Note: Boldface indicate statistical significance

### Somatic mutational landscapes in CRC

NGS data identified somatic mutations in 173 (99.4%) patients of the 174 patients. Fig. S6 shows the top 30 most frequent somatic mutations across all 174 CRC patients. *KRAS* (75/174, 43.1%) and *TP53* (75/174, 43.1%) were the most prominent and significant variations, followed by mutations in *APC* (49/174, 28.2%), *PIK3CA* (41/174, 23.6%), and *GNAS* (35/174, 20.1%).

**Fig. 2:**
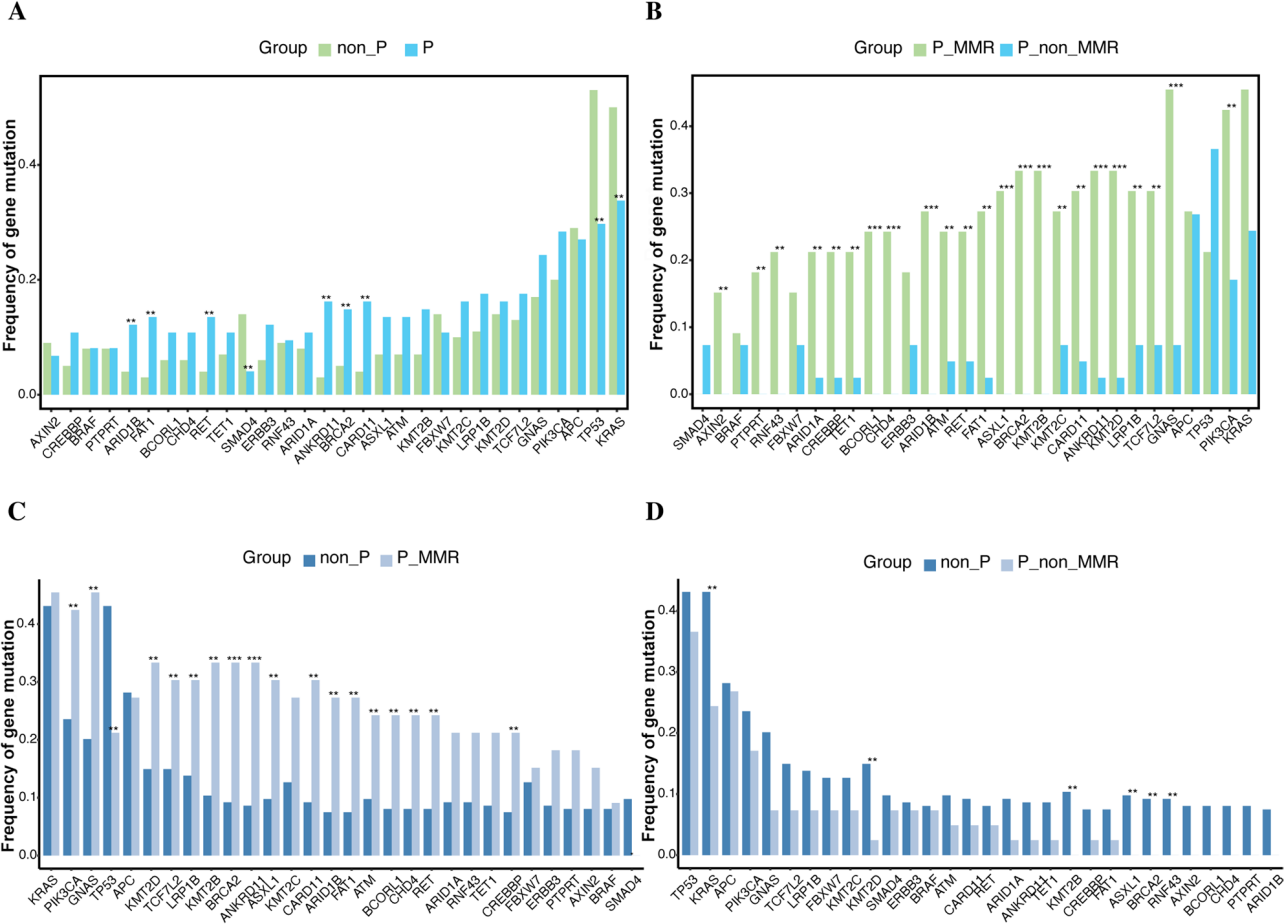
Comparison of somatic mutational frequency of highly mutated genes among the P, non-P, P-MMR, and P-non-MMR groups.** A** Comparison of somatic mutation frequency between patients with and without P/LP germline mutations. **B** Comparison of somatic mutation frequency between patients with and without MMR-related P/LP germline mutations. **C**-**D** Comparison of somatic mutation frequencies in patients without P/LP germline mutations with patients with and without MMR-related P/LP germline mutations, respectively. **P < 0.05; ***P < 0.001

In particular, we focused on the differences in individual mutation frequencies between the P, non-P, P-MMR, and P-non-MMR groups (Figs. 2A-D). Substantial differences in the mutational frequency of highly mutated genes were observed. The P group had a significantly higher mutational frequency in mutations *BRCA2*, *RET*, *ARID1A*, *ARID1B*, *FAT1*, and *CARD11* than in the non-P group (Fig. 2A). In contrast, a higher mutational frequency in genes *KRAS*, *TP53*, and *SMAD4* was found in the non-P group than that in the P group. Notably, significant differences in the frequency of somatic variations were also detected between the P-MMR and P-non-MMR groups (Fig. 2B). The vast majority of genes showed significantly higher mutation frequency in the P-MMR group, whereas *TP53* mutations tended to occur more frequent in the P-non-MMR group.

Next, we investigated the difference among the non-P, P-MMR, and P-non-MMR. The P-MMR group exhibited a significantly higher somatic mutational frequency than the non-P group in genes *PIK3CA*, *GNAS*, *KMT2D*, *TCF7L2*, *LRP1B*, *KMT2B*, *ASXL1*, *ATM*, *BRCA2*, *CARD11*, *ANKRD11*, *BCORL1*, *CHD4*, *RET*, *ARID1B*, *CREBBP*, and *FAT1*. However, *TP53* had a higher mutation frequency in the non-P group than in the P-MMR group (Fig. 2C). Furthermore, patients in the non-P group had a significantly higher frequency of mutations than those in the P-non-MMR group. Specific genes with high mutation rates included *KRAS*, *KMT2D*, *KMT2B*, *ASXL1*, *BRCA2*, and *RNF43* (Fig. 2D). The associations about some genes remained significant in multivariable logistic regression analysis adjusting for age and gender with odds ratio (OR) of 0.515 (0.276–0.949 [95% CI], *P* = 0.033) for *KRAS* mutations; 0.253 (0.133–0.472 [95% CI], *P* < 0.001) for *TP53* mutations; 3.145 (1.125–9.857 [95% CI], *P* = 0.029) for *BRCA2* mutations and 0.327 (0.097–0.903 [95% CI], *P* = 0.030) for *SMAD4* mutations (Table S1).

We further analyzed the mutational signatures using the somatic mutations in both P-MMR and P-non-MMR groups. The distributions of identified mutational signatures were heterogeneous between the two groups, and the mutational signatures enriched in group P-MMR were less abundant than those in group P-non-MMR (Fig. S7). Signature 1, which correlated with age of cancer diagnosis, possessed a higher proportion in both groups. In contrast, signatures 6 and 15 associated with defective DNA MMR became more dominant in the P-MMR group (Fig. S7). These results reveal that distinct mutational processes may be operative between the P-MMR and P-non-MMR groups.

**Fig. 3:**
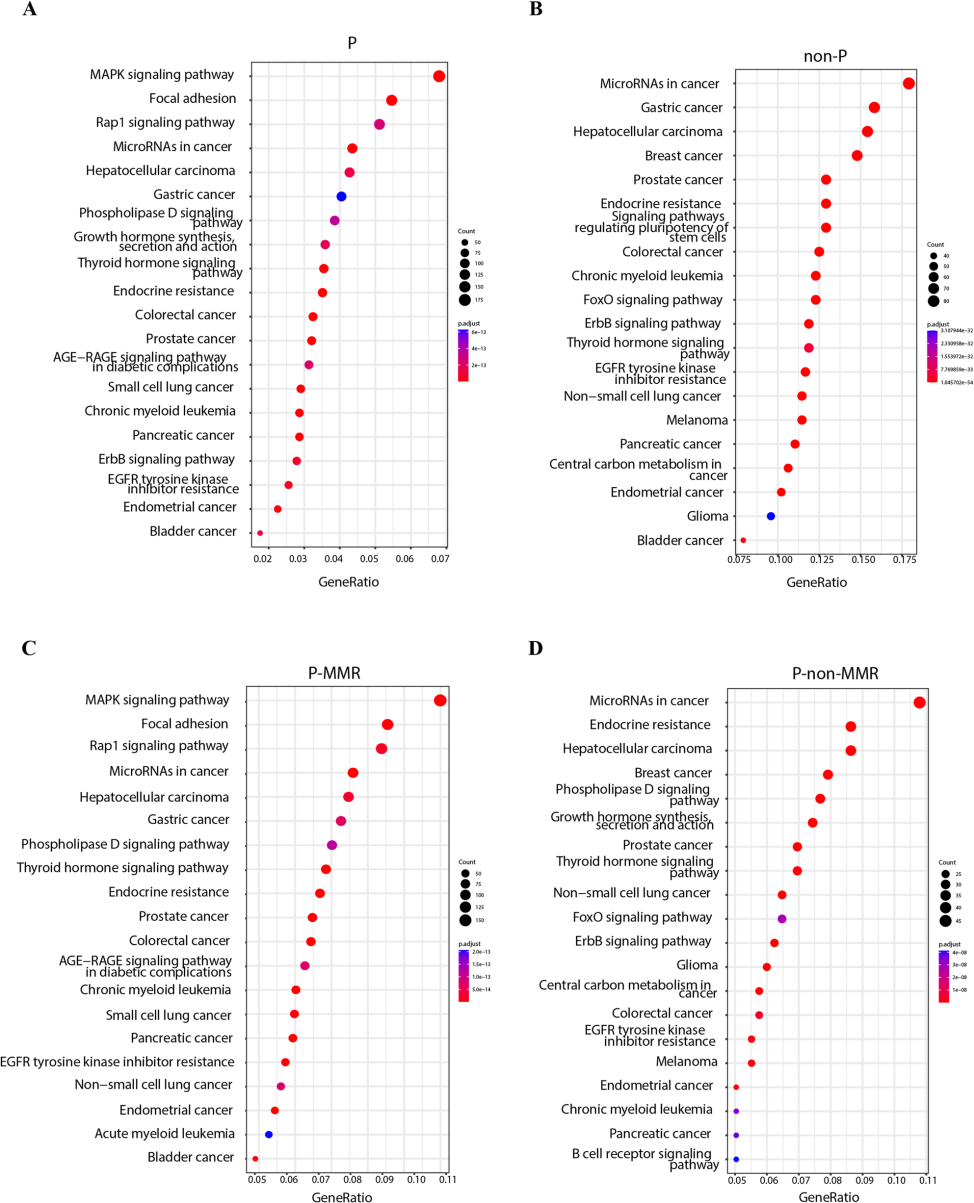
Representative highly significant somatic pathway clustering for the P, non-P, P-MMR, and P-non-MMR groups.** A** KEGG somatic pathway clustering results for patients with P/LP germline mutations. **B** KEGG somatic pathway clustering results for patients without P/LP germline mutations. **C** KEGG somatic pathway clustering results for patients with MMR-related P/LP germline mutations. **D** KEGG somatic pathway clustering results for patients without MMR-related P/LP germline mutations

To delve deeper into the similarities and differences in somatic mutations across the four groups, and explore the mechanistic variations between CRC patients with germline pathogenic variants versus those without, we conducted Kyoto Encyclopedia of Genes and Genomes (KEGG) and gene ontology (GO) clustering analyses. Figure 3 and Figure S8 showed the top 20 most significant clustering in the KEGG and GO analysis for the P, non-P, P-MMR, and P-non-MMR groups. Although the 4 groups of patients had distinct hereditary backgrounds, they shared several common aberrant pathways, thus potentially indicating common carcinogenic mechanisms, including “MicroRNAs in cancer” and “EGFR tyrosine kinase inhibitor resistance” signaling pathways (Fig. 3A-D). In contrast, we observed distinct differences between the P group and the non-P group in terms of biological processes, functions, and pathways (Fig. 3, Fig. S8). The common gene enrichment pathways of the P group include the “MAPK signaling pathway”, “Focal adhesion pathway”, and “Rap1 signaling pathway” which were not found in the non-P group (Fig. 3A-B). Interestingly, the top three enriched signaling pathways in the P-MMR group were consistent with those in the P group; and the P-non-MMR group shared a considerable number of signaling pathways with the non-P group (Fig. 3B and D). Notably, the MAPK signaling pathway was clustered in the P-MMR groups but not the P-non-MMR group (Fig. 3C-D). Additionally, we observed distinct differences across the 4 groups in terms of biological processes, functions, and components, as shown in Fig. S8.

### Germline and somatic gene interactions

Many disease-causing genes in cancer showed strong exclusiveness or co-occurrences in their mutation patterns. We analyzed the distribution of the top 10 germline P/LP variations and the top 20 somatic variations (Fig. S9). The results showed that germline mutations in *BRCA2* were negatively correlated with somatic mutations in *KRAS* (*P* < 0.05). The germline mutation of *MLH1* was positively correlated with the mutations of *PIK3CA*, *CARD11*, *KMT2B*, *TCF7L2*, *RNF43*, *ASXL1*, *ANKRD11*, *ATM* and *BRCA2* (all *P* < 0.01). Notably, germline mutations in *MSH2* were negatively correlated with the *TP53* somatic mutations (*P* < 0.05), and positively correlated with the *LRP1B*, *CARD11*, *KMT2D*, *KMT2B*, *ASXL1*, *ANKRD11*, *KMT2C*, *ARID1A*, *BRCA2*, and *GNAS* somatic mutations (all *P* < 0.01).

### Comparison of MMR gene mutation distributions between our cohort and western cohorts

**Fig. 4:**
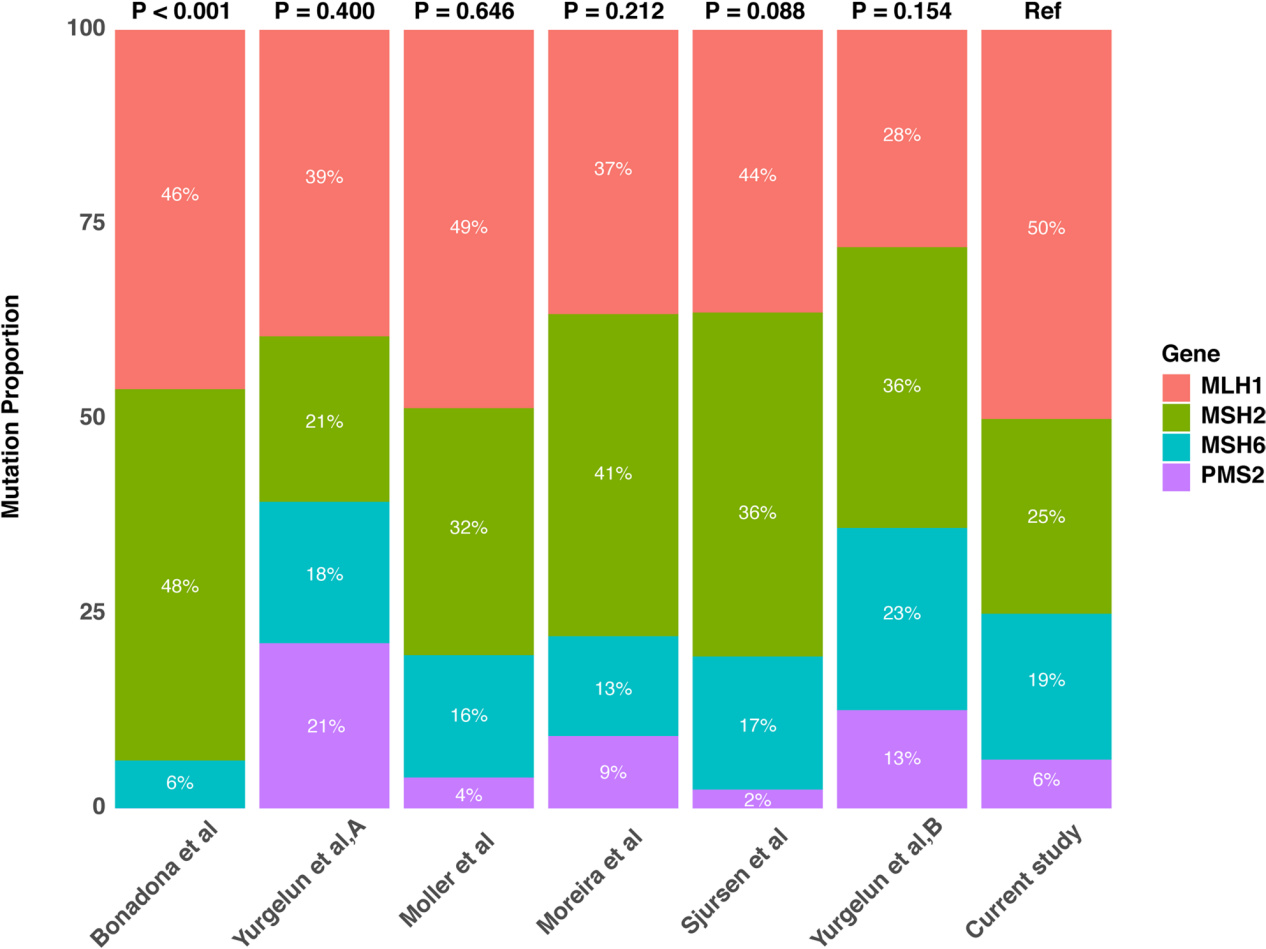
MMR gene mutation distributions compared with prior studies. Fisher’s exact test was used to compare mutation frequencies of MMR genes from this study with those from previous studies. MMR, mismatch repair

Mutation data was collected from patients diagnosed with inherited or family-related CRCs from Western studies [27]. We compared the distribution of mutations in MMR genes (*MLH1*, *MSH2*, *MSH6*, and *PMS2*) between our study participants and those in previously published studies. The result showed that mutation fractions of the four genes differed significantly between our study and those reported by Bonadona et al. (Fig. 4) [19]. Unlike the majority of Western cohorts, our Chinese dataset revealed that patients with *MSH2* mutations, which are significant in the hereditary risks of LS [20], constituted a lower proportion (25%).

### Germline mutations increase the risk of Chinese CRC patients

Germline mutations may elevate the likelihood of developing cancer. To assess the increased risk of CRC in individuals with these mutations, we calculated the ORs for each specific germline mutation and all mutations collectively. The frequency of germline mutations within the general population was ascertained through gnomAD screening. Utilizing this data, we calculated the OR for each mutation site, and for all mutations combined, by contrasting the prevalence in the general population with the mutation frequency identified in our study, serving as a gauge of CRC risk. In Table 3, we present comprehensive demographic and mutational data, along with the calculated ORs for pathogenic mutations. These findings provide compelling evidence of a substantial increase in pathogenic mutations, especially *BRCA2* and *ATM*, within the studied population of CRC patients. Consequently, individuals harboring these germline mutations may be at a significantly elevated risk of developing CRC.

**Table 3:** Pathogenic germline mutations identified in this study

Gender	Age	Hugo Symbol	HGVSp	HGVSc	VAF	Allele count	Allele frequency in CRC ^[1]^	Allele count	Allele frequency in general population ^[2]^	Variant ID	Clinvar significance	OR	95% CI	*P *value
Male	NA	ATM	p.R2034Ter	c.6100 C > T	0.571	1	5.76E-05	1	4.06E-06	127,417	LP	14.18904679	0.1807–1102	0.127
Male	71	ATM	p.Q1098Ter	c.3292 C > T	0.474	1	5.76E-05	1	4.21E-06	938,009	P	13.69470826	0.1745–1065	0.131
Female	74	BRCA1	p.R1699Q	c.5096G > A	0.495	1	5.76E-05	1	2.44E-05	37,636	P	2.363791267	0.05139–19.48	0.379
Male	33	BRCA2	p.Q1037Ter	c.3109 C > T	0.495	2	0.00011526	2	4.09E-06	37,819	P	28.18126053	1.466–1641	0.013
Male	65	BRCA2	p.Q1037*	c.3109 C > T	0.294	2	0.00011526	2	4.09E-06	37,819	P	28.18126053	1.466–1641	0.013
Male	66	BRIP1	p.W448Ter	c.1343G > A	0.422	1	5.76E-05	1	2.48E-05	216,129	LP	2.320547063	0.05045–19.13	0.385
Female	52	MRE11	NA	c.659 + 1G > A	0.399	1	5.76E-05	1	2.04E-05	230,014	LP	2.829643443	0.05982–25.28	0.336
Male	54	MRE11	p.R364Ter	c.1090 C > T	0.401	1	5.76E-05	1	4.47E-05	140,953	P	1.289642355	0.02996–8.875	0.558
Female	48	MSH2	p.R711Ter	c.2131 C > T	0.754	1	5.76E-05	1	3.23E-05	90,903	P	1.783699606	0.02272–139.9	1.000
Male	54	MSH6	p.R1076C	c.3226 C > T	0.471	1	5.76E-05	1	9.75E-05	89,357	LP	0.590952558	0.01437–3.625	1.000
Male	65	RAD51C	NA	c.905–2 A > C	0.431	1	5.76E-05	1	8.13E-06	216,132	LP	7.092685364	0.1202–136.3	0.185
Male	52	RET	p.V804M	c.2410G > A	0.453	1	5.76E-05	1	0.000126701	37,102	LP	0.45482326	0.01114–2.743	0.721
Female	48	SDHA	p.M1V	c.1 A > G	0.340	1	5.76E-05	1	8.57E-06	239,661	LP	6.72840257	0.0857–525.7	0.242
Male	50	TP53	p.P152L	c.455 C > T	0.491	1	5.76E-05	1	8.13E-06	142,766	P	7.091474678	0.1202–136.3	0.185

*VAF* variant allele frequency, *OR* odds ratio

^[1]^ Data from current study

^[2]^ Data from gnomAD

## Discussion

Previous studies have established associations between P/LP germline mutations and hereditary CRC and just in some common genes, such as *APC*, *MLH1*, *MSH2*, *MSH6*, and *PMS2* [28, 29]. With the development of NGS technology, more and more germline mutations have been discovered, and investigating the distribution of germline mutations, including rare genes, and their contributions to the pathogenesis of CRC is a topic deserving of scientific inquiry [30]. We utilized multigene panel testing to investigate the prevalence and variety of germline mutations in genes associated with various hereditary cancer syndromes in a cohort of 8676 Chinese CRC patients unselected. We discovered that eighty patients carried pathogenic germline mutations, primarily in high-penetrance cancer susceptibility genes. Additionally, we thoroughly analyzed somatic mutations in the Chinese cohort. Our research sheds light on the genetic factors contributing to Chinese CRC.

A significantly higher proportion of patients in P group had an age < 50 years compared to non-P patients, consistent with previous findings [31], confirming the early onset of CRC in patients with P/LP mutations. This finding underscores the importance of genetic testing for identifying individuals at higher risk for developing CRC at a younger age. Yarchoan et al., Margalit et al., and their colleague have demonstrated that patients with TMB-H and MSI-H tumors can derive significant benefits from immunotherapy [32–34]. The higher proportion of MSI-H patients in the P group suggests that these patients may be particularly responsive to immunotherapy, highlighting the potential for personalized treatment approaches based on genetic profiling. Interestingly, we observed a higher ratio of TMB-H and MSI-H tumors in the P-MMR group compared to the P-non-MMR group. This finding may inform the optimization of subsequent screening and treatment strategies, thereby maximizing the clinical benefits of immunotherapy in patients harboring P/LP germline mutations. These findings emphasize the value of genetic testing and molecular profiling in identifying patients who may benefit most from targeted therapies, ultimately improving outcomes and personalized care for CRCs.

Some common germline mutations have garnered significant clinical interest due to their association with hereditary cancer syndromes. For example, LS is a well-described hereditary cancer syndrome caused by germline mutations in MMR genes (*MLH1*, *MSH2*, *MSH6*, and *PMS2*) or by a deletion in the *EPCAM* gene [35, 36]. FAP is caused by germline mutations in the *APC* gene [8]. Although only a small proportion of these polyps’ progress along the adenoma-adenocarcinoma sequence, this still results in an almost 100% cumulative lifetime risk of CRC [37]. Biallelic germline mutations of the base-excision-repair gene MUTYH resulted MAP [38], which contribute to a considerable proportion of attenuated forms of colorectal adenomatous polyposis. Individuals with monoallelic pathogenic MUTYH variants and a positive family history of CRC have been reported to have a twofold increased risk of CRC and extracolonic cancers [39]. While *PTEN* mutations are associated with PTEN hamartoma tumor syndrome. Additionally, mutations in *STK11* are found in Peutz-Jeghers syndrome, and *SMAD4*/*BMPR1A* mutations in juvenile polyposis syndrome. The identification of new germline variants is crucial for pinpointing founding mutations, and NGS techniques have revolutionized the detection of such variants. In our research, we detected germline mutations from 21 genes, including some rare genes, such as *FH* and *SDHA*. The presence of these rare mutations may indicate an increased risk of CRC in carriers, underscoring the importance of personalized guidance and surveillance for individuals with such mutations. By expanding our understanding of germline mutations, we can enhance risk assessment and tailor preventive strategies for at-risk individuals.

CRC is a heterogeneous disease, with a wide range of somatic mutations playing a crucial role in its development and progression [40]. By examining the distribution and variations of these mutations, researchers can gain valuable insights into the underlying mechanisms of carcinogenesis. We focused on comparing the landscape of somatic mutations based on the pathogenicity classification of germline mutations. Our analysis revealed that genes with high mutation rates in the P group exhibited significantly higher mutation frequencies than those in the non-P group. This observation suggests that mutations in the P group may impact key pathways involved in DNA repair, such as MMR, DNA damage response, and homologous recombination, leading to an accumulation of somatic mutations. Specifically, the P MMR-related group showed a greater number of mutations across multiple genes, indicating potential implications for immunotherapy response and patient prognosis. Interestingly, we also identified differences in the mutational landscape between the P and non-P groups concerning specific genes. For instance, mutations in *KRAS* and *TP53* were more frequently observed in the non-P group, highlighting distinct molecular characteristics that may influence disease progression and treatment outcomes. Interactions analysis can help identify the tumor’s hetero-mutated genes and can help define the tumor subtype. We also found some interactions between germline and somatic genes. For example, the germline mutation of *MLH1* was positively correlated with the somatic mutation of *PIK3CA* and *BRCA2*, and germline mutations in *MSH2* were negatively correlated with the *TP53* somatic mutations and positively correlated with the *BRCA2* somatic mutations. By elucidating the intricate interplay of somatic mutations in different genetic contexts, we aim to advance personalized treatment strategies and improve clinical outcomes for patients with this challenging disease.

The analysis of KEGG pathways showed that the four groups of patients with varying hereditary backgrounds exhibited shared aberrant pathways, such as the involvement of MicroRNAs in cancer and EGFR tyrosine kinase inhibitor resistance signaling pathways. These common pathways may suggest underlying carcinogenic mechanisms that are consistent across different patient groups. One particularly significant pathway identified was the MAPK pathway, a complex signaling network that plays a crucial role in regulating various cellular processes including growth, differentiation, apoptosis, and oncogenic transformations [41]. Interestingly, the MAPK pathway was found to be significantly enriched in patients of the P group and patients of the P group with MMR-related germline mutations. This discrepancy highlights potential differences in oncogenic signaling pathways between MMR and non-MMR mutation-driven CRCs, suggesting tailored therapeutic strategies may be necessary. This observation indicates potential differences in the pathogenesis of CRC between patients with germline mutations and those without, including individuals with LS. Identifying MAPK pathway activity in germline mutant CRC patients highlights the importance of understanding the distinct mechanisms underlying the development of CRC in different patients. This insight contributes to our understanding of the molecular basis of CRC and provides valuable information for molecular subtyping and personalized treatment strategies. By elucidating these molecular pathways, our study offers valuable insights that can potentially improve the management and treatment outcomes for patients with CRC.

The frequency of MMR gene mutations differs between Chinese and Western patients. Our findings indicate a lower prevalence of *MSH2* mutations among Chinese patients than Western counterparts, consistent with an earlier report [27]. Genetic diversity between populations may play a role in influencing the prevalence of specific mutations. Other factors may also contribute to this discrepancy, such as healthcare access and screening practices. Further research is critical to elucidate these factors and their implications for clinical management and genetic counseling in diverse populations.

Finally, our research delved into quantifying the risk associated with P/LP germline mutations in CRC through the calculation of OR. The prevalence of mutations identified through gnomAD screening reflects the occurrence of specific genetic changes within the broader population, and this approach has been used in multiple studies [42–44]. When it comes to more common germline mutations, it becomes feasible to determine the risk associated with individual genes. However, there is a pressing need for extensive population studies and familial data to draw concrete conclusions about rarer gene mutations. In our study, these high OR values reinforce the role of *ATM* and *BRCA2* as high penetrance genes for CRC susceptibility, warranting their inclusion in genetic screening panels. Germline pathogenic variants ATM and BRCA2 carriers with breast cancer were at significantly elevated risk of contralateral breast cancer [45–47]. Several well-known predisposition genetic variants including BRCA2 have been found to have strong association with lung cancer risk PMID: 24,880,342. BRCA1/2 germline mutations have also been reported in CRC [48]. All BRCA1/2 P germline mutations reported herein are associated with CRC, on the basis of clear clinical evidence. The pathogenic variants of BRCA2 (OR = 1.9) were significantly associated with CRC development in the Japanese population, and were significantly associated with diagnosis age and personal/family history of cancer [49]. The work by Garre et al. have provided the first evidence of germline BRCA2 pathogenic mutation associated with CRC risk [50]. P/LP germline mutations in the HRR and CPF genes, such as BRCA1/2 were associated with moderately to highly elevated CRC risks [51]. All findings suggest that BRCA2 germline mutation was associated with CRC risk.

These findings highlight the importance of genetic screening and counseling for individuals with a family history of CRC or a suspected genetic predisposition. Identifying at-risk individuals through genetic testing could facilitate early detection, develop personalized treatment strategies, and improve outcomes for those at increased risk of developing CRC. Further research and collaboration are essential to improve our understanding of the genetic factors that influence CRC risk and to develop targeted interventions for at-risk populations.

Our study has some limitations. Being a retrospective study, there is a possibility of selection bias in the patient cohort, which could impact the generalizability of our findings. Additionally, the sample size was relatively limited, and the lack of comprehensive clinical data, particularly prognostic information and additional risk markers, hinders a more thorough analysis of the results. Further prospective studies with larger and more diverse cohorts are essential to validate the generalizability of these findings and refine hereditary CRC risk models. Despite these limitations, our study has made significant contributions by utilizing multigene panel testing to uncover several rare germline mutations, including *FH* and *SDHA*, which have not been extensively documented in the context of inherited CRC.

## Conclusions

Our study represents a comprehensive analysis of germline and somatic mutations in CRC by multigene panel testing. The distribution of germline mutations in CRC was fully demonstrated and the cancer risk of different germline mutations was assessed. Our findings may help gain insights into the genetic basis of CRC and generate accurate individualized risk management strategies for mutation carriers.

## Supplementary Information

Supplementary Material 1.

## Data Availability

The datasets generated during and/or analyzed during the current study are available from the corresponding author upon reasonable request.
