# Supplementary material for: Genetic landscape of Chinese colorectal cancer: insights into germline and somatic mutations
Source: BMC Cancer. 2026 Mar 10;26:492. doi: 10.1186/s12885-026-15626-x (PMC13088694; doi:10.1186/s12885-026-15626-x)
Supplement: Supplementary file 1 — Supplementary Material 1. [file 12885_2026_15626_MOESM1_ESM.docx]

**Supplementary for**

**Genetic Landscape of Chinese Colorectal Cancer: Insights into Germline and Somatic Mutations**

Fazhi Zhao^#1^, Hexin Lin^#2^, Rui Han^#3^, Meng Wang^#4^, Lin Yang^5^, Dianfeng Tian^6^, Pengxin Zhang^*7^, Queling Liu^*8, 9^

^1^ Department of Gastric Surgery, Sichuan Clinical Research Center for Cancer, Sichuan Cancer Hospital and Institute, Sichuan Cancer Center, Affiliated Cancer Hospital of University of Electronic Science and Technology of China, Chengdu, Sichuan Province, 610041, China

^2^ Department of Gastrointestinal Oncology Surgery, The First Affiliated Hospital of Xiamen University, School of Medicine, Xiamen University, Xiamen, Fujian, 361001, China

^3^ Department of Pathology, Peking University First Hospital, Beijing, 100034, China

^4^ Center of Gastrointestinal and Minimally Invasive Surgery, Department of General Surgery, The Third People’s Hospital of Chengdu, Chengdu, 610031, China

^5^ Department of urology, Aerospace center hospital, Beijing, 100049, China

^6^ Department of Colorectal Surgery, First Hospital of Shanxi Medical University, Taiyuan, Shanxi, 030001, China

^7^ Department of Pathology, The First Affiliated Hospital of Dalian Medical University, Dalian, Liaoning Province, 116011, China

^8^ Department of Oncology, The Second Affiliated Hospital of Nanchang University, 330000 Nanchang, Jiangxi, China

^9^ Department of Oncology, Jiangxi Provincial People’s Hospital, The First Affiliated Hospital of Nanchang Medical College, 330000 Nanchang, Jiangxi, China

# Fazhi Zhao, Hexin Lin, Rui Han and Meng Wang contributed equally to this work.

**Correspondence**

Pengxin Zhang, Department of Pathology, The First Affiliated Hospital of Dalian Medical University, Dalian, Liaoning Province, 116011, China. Email: zhangpengxin1983@163.com.

Queling Liu, ^1^ Department of Oncology, The Second Affiliated Hospital of Nanchang University, 330000 Nanchang, Jiangxi, China; ^2^ Department of Oncology, Jiangxi Provincial People’s Hospital, The First Affiliated Hospital of Nanchang Medical College, 330000 Nanchang, Jiangxi, China. Email: 71537430@qq.com.

**Guarantor of the article:** Queling Liu

**This file includes:**

**Supplementary Figures: S1 to S9**

**Supplementary Tables: S1**

**
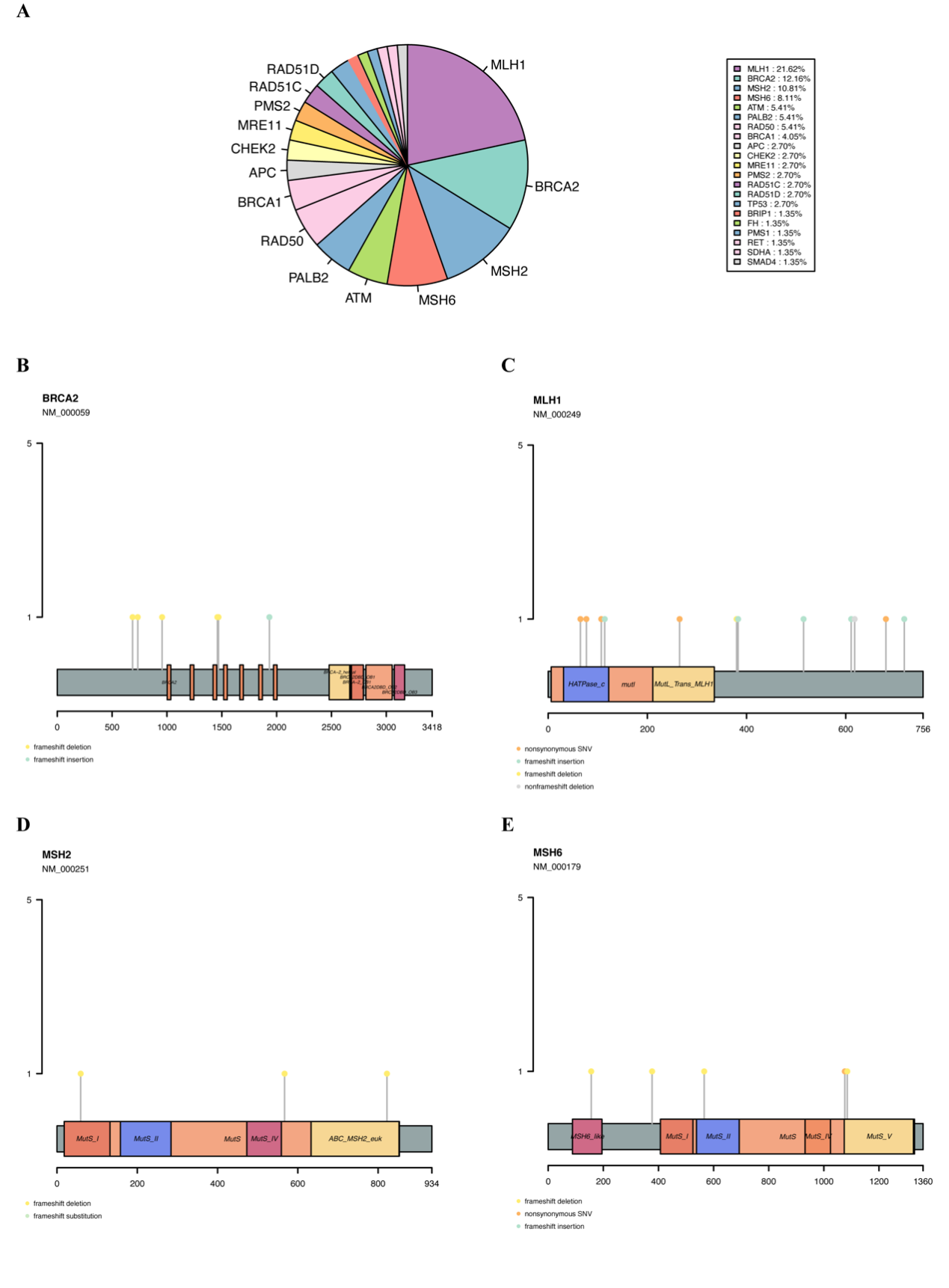
**

**Figure S1 The germline mutation distributions and mutant sites of the top 4 genes harboring high-frequency mutations in CRC. (A)** The pie chart shows the distribution of germline mutations. **(B)** Distributions of *BRCA2* mutations identified in CRC. **(C)** Distributions of *MLH1* mutations identified in CRC. **(D)** Distributions of *MSH2* mutations identified in CRC. **(E)** Distributions of *MSH6* mutations identified in CRC.

**
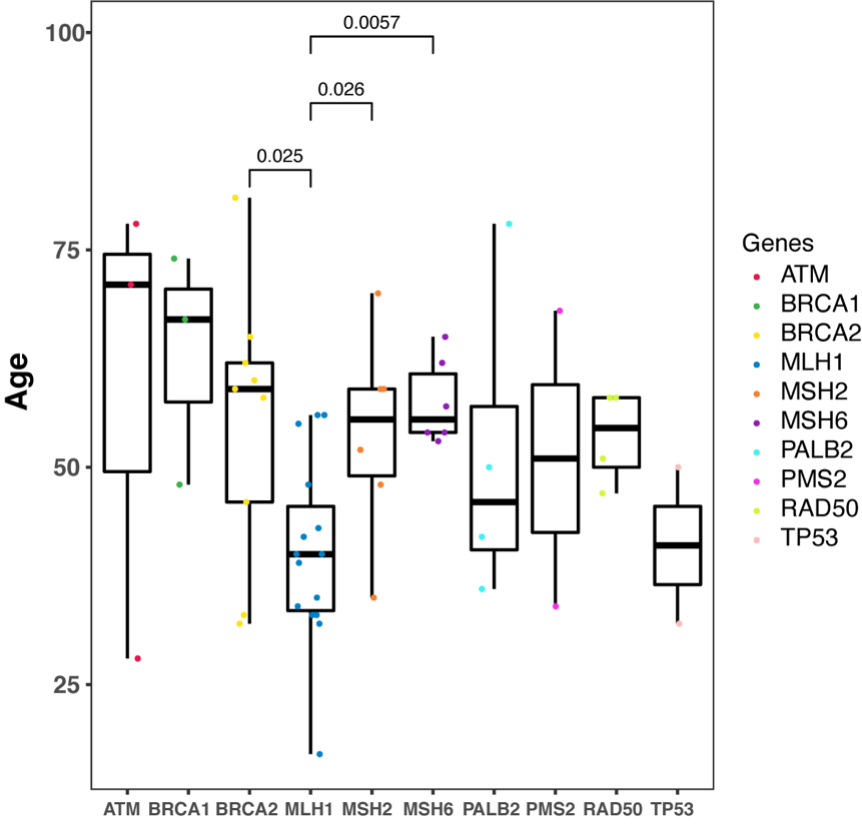
**

**Figure S2 The age distribution at diagnosis of the 74 patients with different germline mutations.**


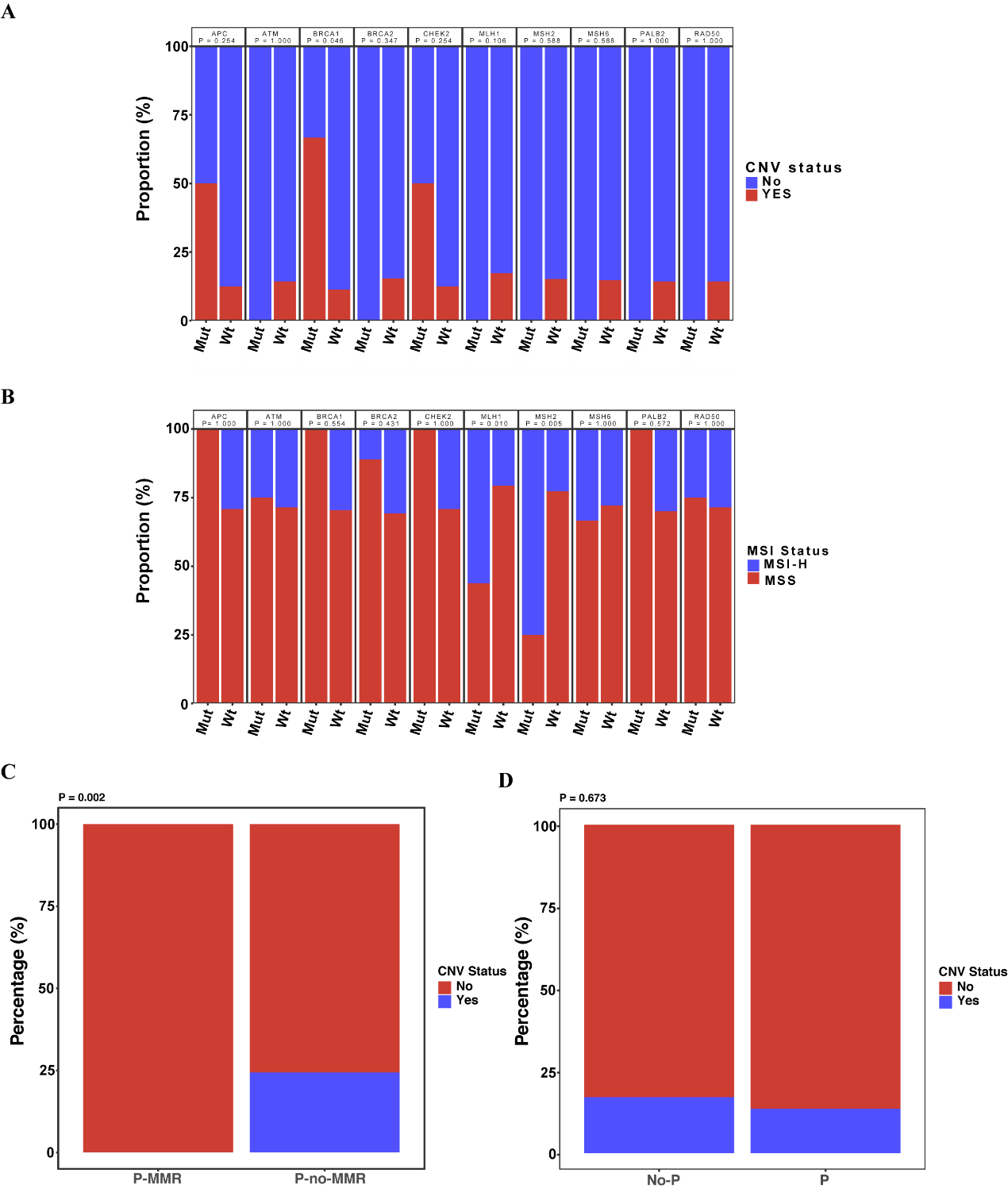


**Figure S3 CNV status and MSI distribution in patients with different germline gene mutations.** (**A**) CNV status in patients with different germline gene mutations. (**B**) MSI distribution in patients with different germline gene mutations. (**C**) CNV status in patients with or without germline gene mutations. (**D**) CNV status in patients with or without MMR gene germline mutations.

**
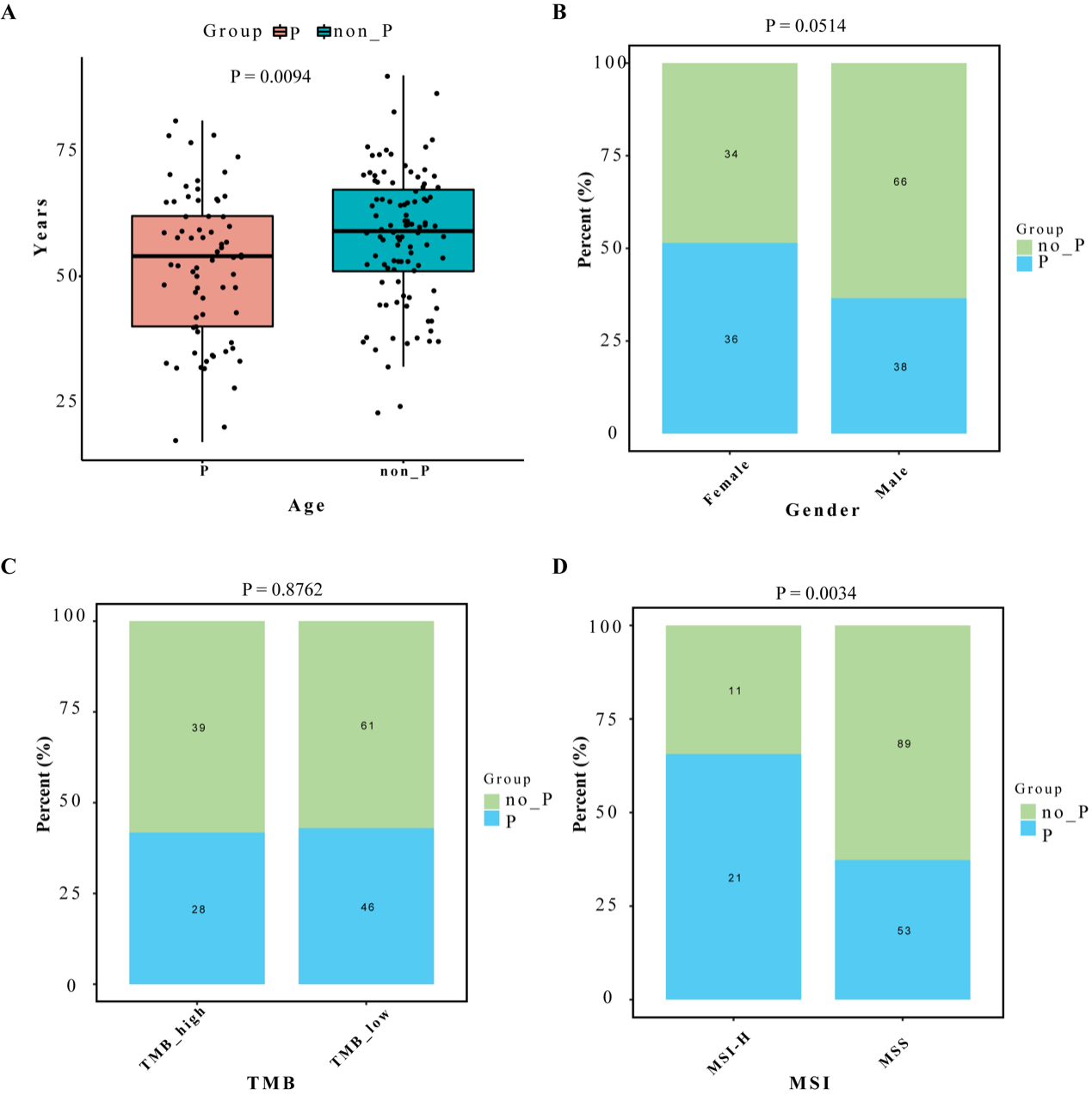
**

**Figure S4 Comparison of clinical characteristics between patients with and without P germline mutations, including age (A), gender (B),** **TMB (C), and MSI (D).** TMB, tumor mutation burden; MSI, microsatellite instability; MSI-H, microsatellite instability-high; MSS, microsatellite stable.

**
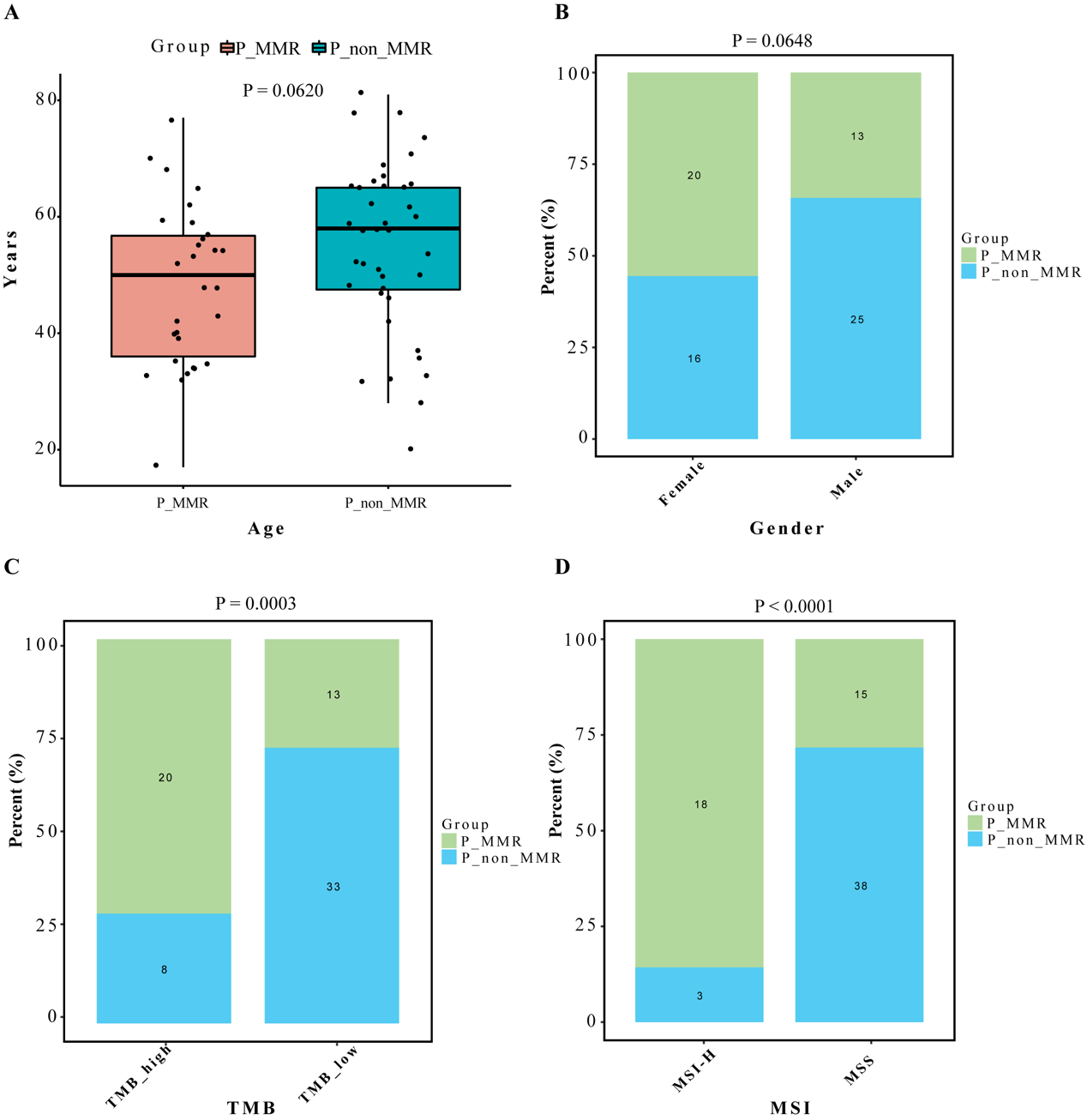
**

**Figure S5 Comparison of clinical characteristics** **between patients with and without MMR-related P germline mutations, including age (A), gender (B), TMB (C), and MSI (D).** TMB, tumor mutation burden; MSI, microsatellite instability; MSI-H, microsatellite instability-high; MSS, microsatellite stable.


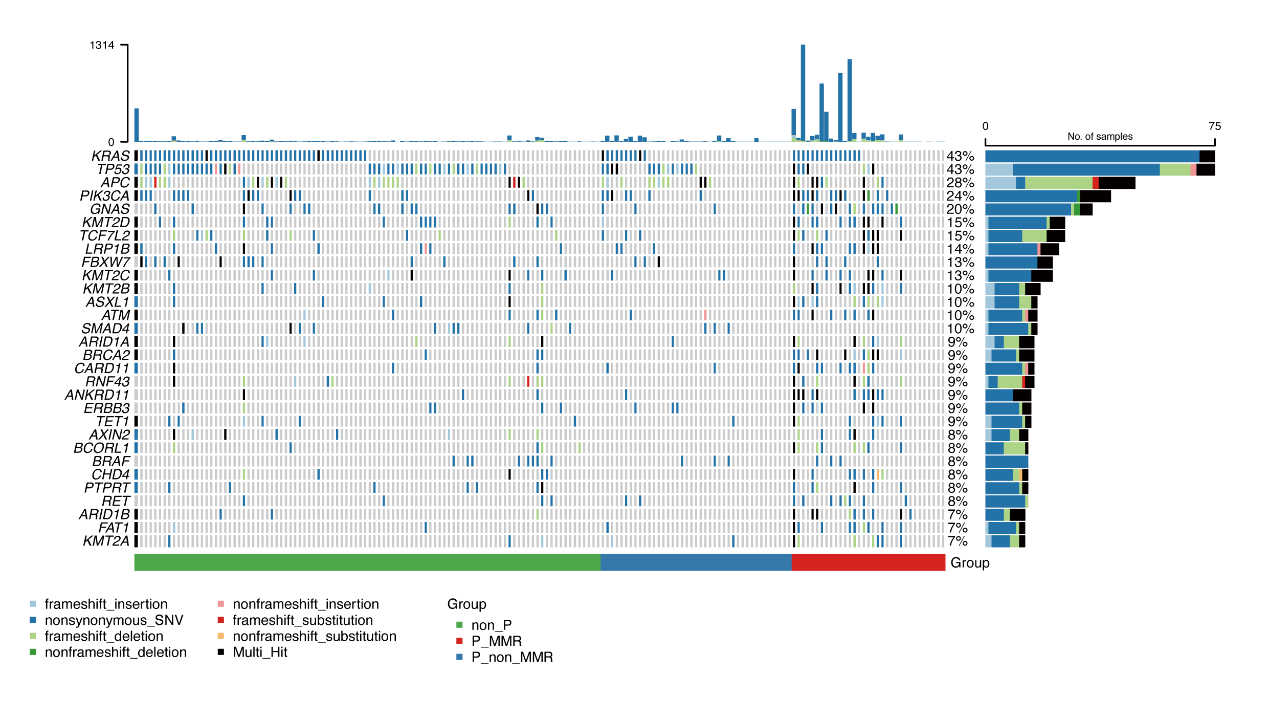


**Figure S6 Somatic mutation landscape of the 174 CRC.** Heatmap illustrating the top 30 genes identified in our study. Each column represents one patient, and each row represents an alternation. The upper bars represent the total mutations. The right bars indicate the frequency of mutated genes.

**
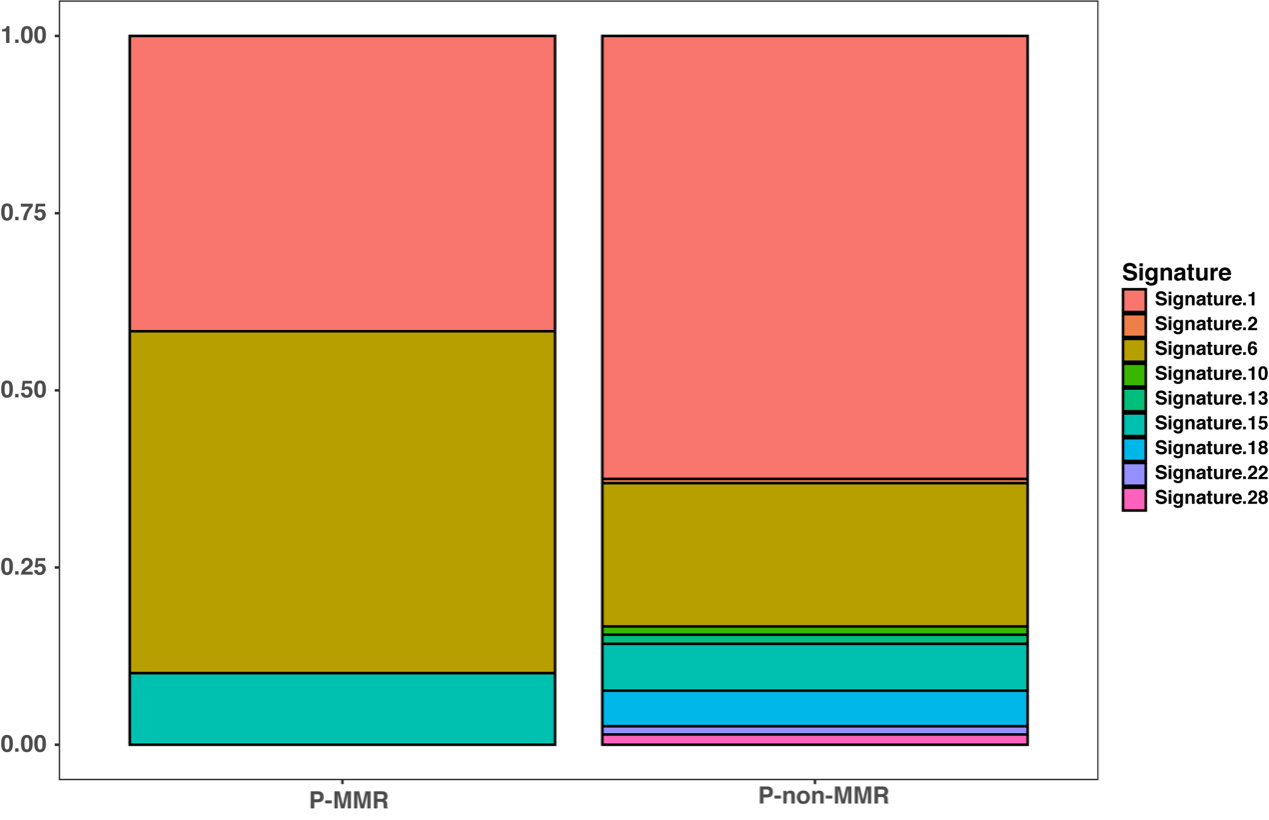
**

**Figure S7** **Analysis of mutational signatures between patients with and without MMR-related P germline mutations.**


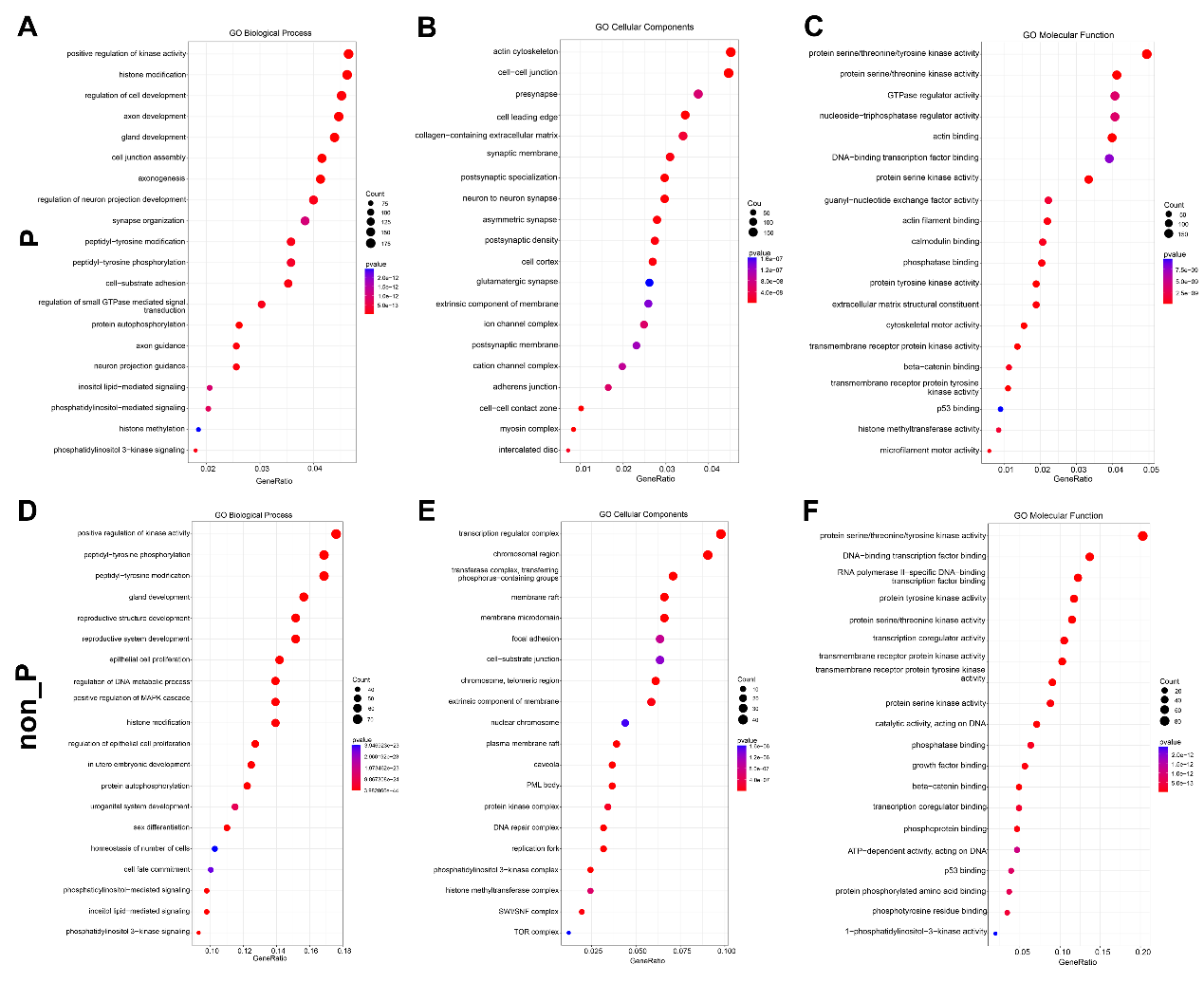


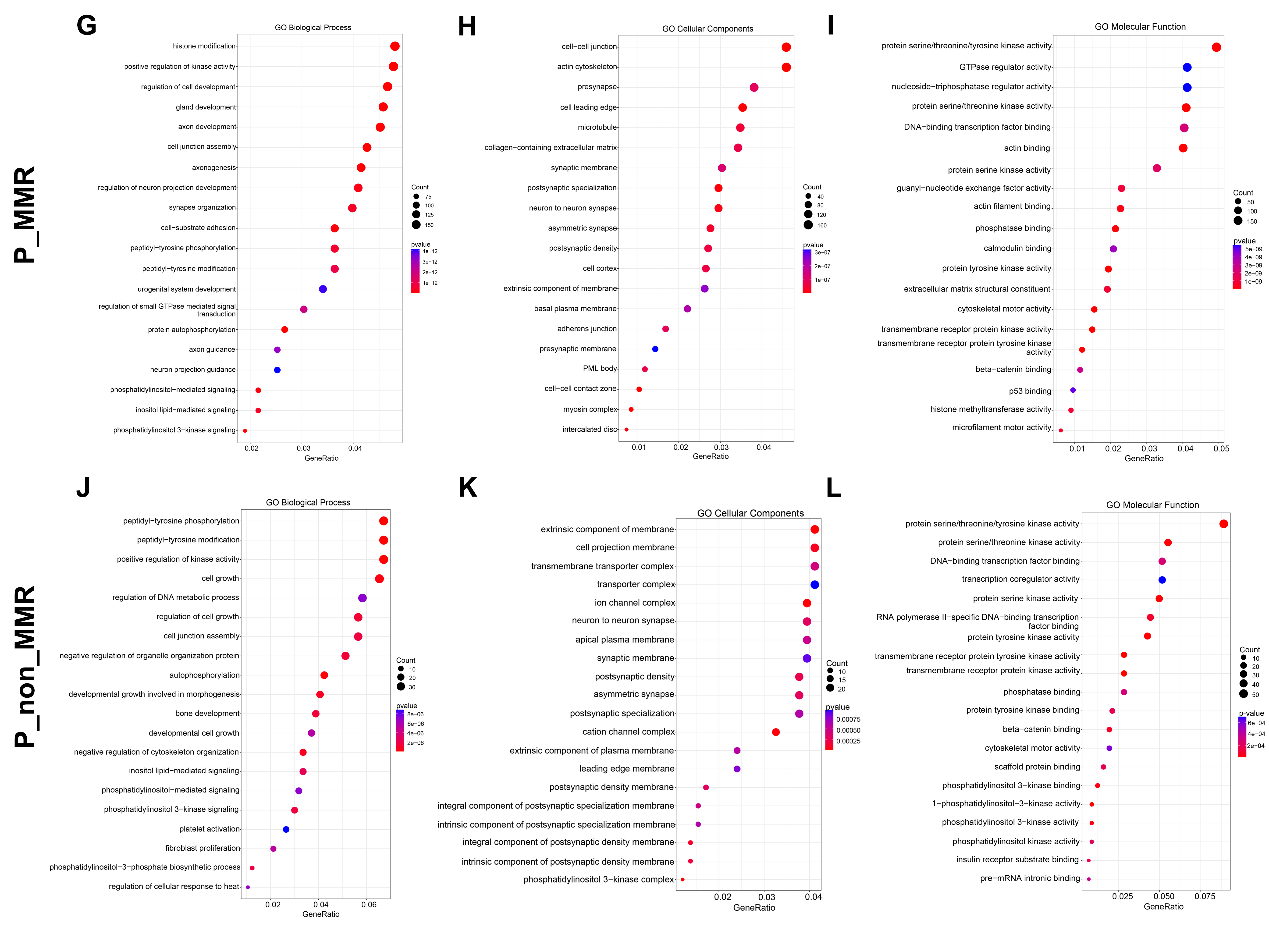


**Figure S8 Representative highly significant somatic pathway clustering for the P, non-P, P-MMR, and P-non-MMR groups. A-C** GO somatic pathway clustering results for patients with P germline mutations (BP, CC, and MF, respectively). **D-F** GO somatic pathway clustering results for patients without P germline mutations (BP, CC, and MF, respectively). **G-I** GO somatic pathway clustering results for patients with MMR-related P germline mutations (BP, CC, and MF, respectively). **J-L** GO somatic pathway clustering results for patients without MMR-related P germline mutations (BP, CC, and MF, respectively). BP, biological process; CC, cellular components; MF, molecular function.

**
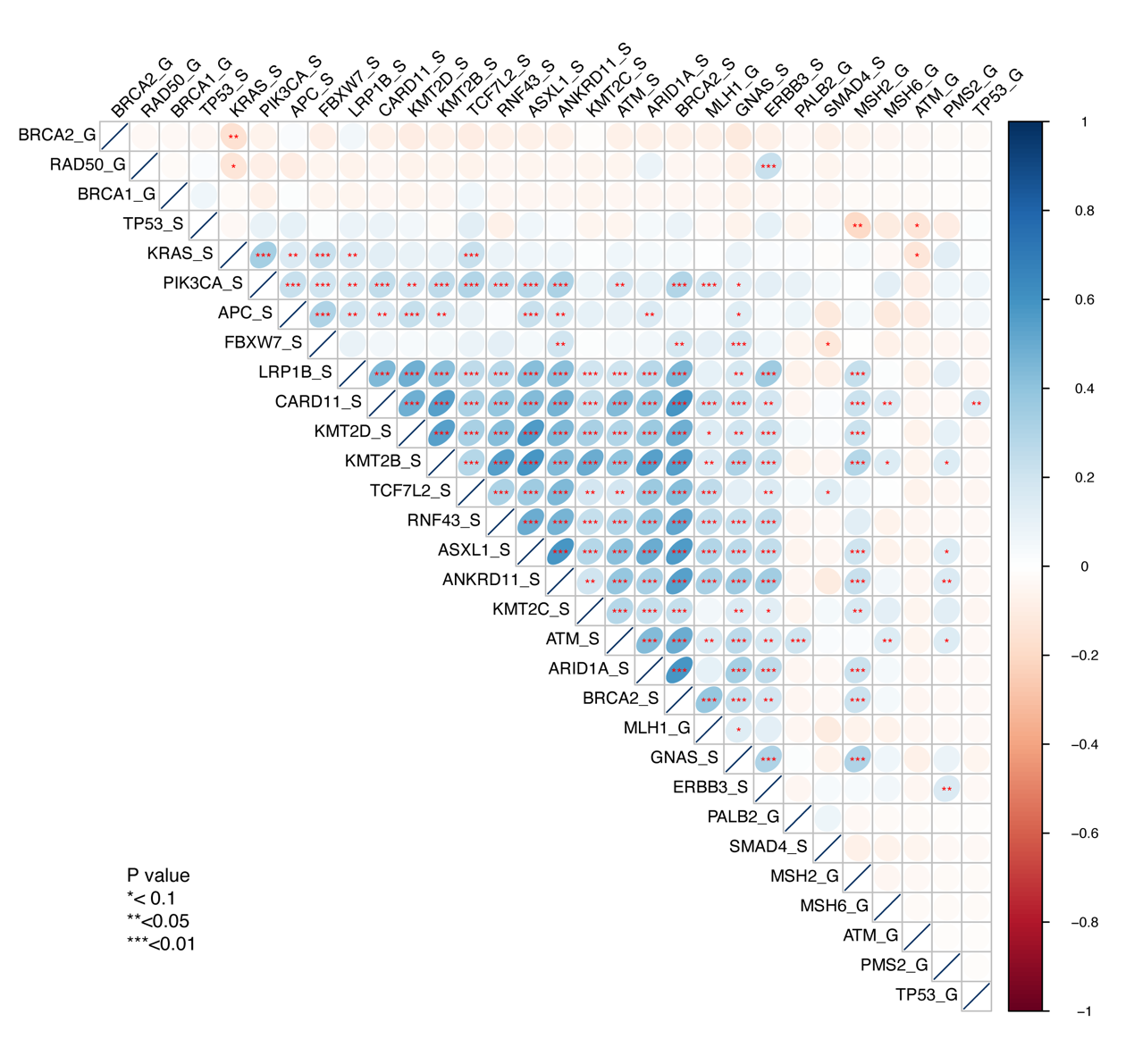
**

**Figure S9 Mutual interactions analysis of the selected genes.** S, somatic mutations, and G, pathogenic germline mutations. **P* < 0.1; ***P* < 0.05; ****P* < 0.01.**Table S1 Correlation between germline mutation status and somatic mutations.**

|  | **Univariable analysis** | |  | **Multivariab analysis** | |
| --- | --- | --- | --- | --- | --- |
|  | **OR (95% CI)** | ***P*** value |  | **OR (95% CI)** | ***P*** value |
|  | *KRAS* mutation |  |  |  |  |
| Age | 1.004(0.982-1.026) | 0.743 |  | 1.001(0.979-1.025) | 0.918 |
| Gender (Male) | 0.838(0.456-1.54) | 0.568 |  | 0.766(0.401-1.456) | 0.416 |
| Germline mutation (Positive) | 0.515(0.276-0.949) | **0.033** |  | 0.525(0.274-0.992) | **0.047** |
| TMB (Low) | 0.368(0.195-0.684) | **0.001** |  | 0.315(0.152-0.633) | **0.001** |
| MSI status | 0.828(0.387-1.785) | 0.626 |  | 1.097(0.444-2.761) | 0.842 |
|  | *TP53* mutation |  |  |  |  |
| Age | 1.009(0.988-1.032) | 0.401 |  | 1(0.976-1.024) | 1.000 |
| Gender (Male) | 1.314(0.715-2.417) | 0.379 |  | 1.065(0.542-2.073) | 0.854 |
| Germline mutation (Positive) | 0.253(0.133-0.472) | **<0.001** |  | 0.29(0.149-0.553) | **<0.001** |
| TMB (Low) | 0.755 (0.404-1.394) | 0.370 |  | 0.403(0.175-0.871) | **0.020** |
| MSI status | 3.036(1.398-6.894) | **0.005** |  | 3.636(1.416-9.985) | **0.007** |
|  | *BRCA2* mutation |  |  |  |  |
| Age | 0.97(0.932-1.008) | 0.123 |  | 0.979(0.939-1.02) | 0.305 |
| Gender (Male) | 0.498(0.176-1.365) | 0.174 |  | 0.705(0.217-2.284) | 0.554 |
| Germline mutation (Positive) | 3.145(1.125-9.857) | **0.029** |  | 1.991(0.636-6.652) | 0.236 |
| TMB (Low) | 0.014(0.0001-0.111) | **<0.001** |  | 0.038(0.0002-0.331) | **<0.001** |
| MSI status | 0.036(0.009-0.117) | **<0.001** |  | 0.094(0.016-0.414) | **0.001** |
|  | *SMAD4* mutation |  |  |  |  |
| Age | 0.99(0.957-1.024) | 0.553 |  | 0.983(0.948-1.019) | 0.352 |
| Gender (Male) | 0.64(0.254-1.614) | 0.340 |  | 0.631(0.235-1.712) | 0.361 |
| Germline mutation (Positive) | 0.327(0.097-0.903) | **0.030** |  | 0.227(0.055-0.703) | **0.009** |
| TMB (Low) | 0.916(0.365-2.399) | 0.854 |  | 0.640(0.226-1.879) | 0.408 |
| MSI status | 10.878(1.426-1396) | **0.015** |  | 9.994(1.160-1318) | **0.033** |

OR, odds ratio; CI, confidence interval.
